# P4HA2 activates mTOR via hydroxylation and targeting P4HA2-mTOR inhibits lung adenocarcinoma cell growth

**DOI:** 10.1038/s41388-024-03032-1

**Published:** 2024-04-23

**Authors:** Ersuo Jin, Shengjie Wang, Donglai Chen, Jia-Ping Wang, Yuanyuan Zeng, Runfeng Sun, Hong-Tao Zhang

**Affiliations:** 1 Soochow University Laboratory of Cancer Molecular Genetics, Collaborative Innovation Center of Molecular Medicine between Soochow University and Donghai County People’s Hospital, Suzhou Medical College of Soochow University, Suzhou, 215123 Jiangsu Province China; 2 Department of Genetics, School of Basic Medical Sciences, Suzhou Medical College of Soochow University, Suzhou, 215123 Jiangsu Province China; 3 Department of Basic Medicine, Kangda College of Nanjing Medical University, Lianyungang, 222000 Jiangsu Province China; 4 Department of Thoracic Surgery, Zhongshan Hospital, Fudan University, Shanghai, 200032 China; 5 Donghai County People’s Hospital, Lianyungang, 222000 Jiangsu Province China; 6 Department of Respiratory Medicine, The First Affiliated Hospital of Soochow University, Suzhou Medical College of Soochow University, Suzhou, 215006 Jiangsu Province China; 7 Suzhou Key Laboratory for Molecular Cancer Genetics, Suzhou, 215123 Jiangsu Province China

**Keywords:** TOR signalling, Post-translational modifications

## Abstract

Mammalian target of rapamycin (mTOR) kinase functions as a central regulator of cell growth and metabolism, and its complexes mTORC1 and mTORC2 phosphorylate distinct substrates. Dysregulation of mTOR signaling is commonly implicated in human diseases, including cancer. Despite three decades of active research in mTOR, much remains to be determined. Here, we demonstrate that prolyl 4-hydroxylase alpha-2 (P4HA2) binds directly to mTOR and hydroxylates one highly conserved proline 2341 (P2341) within a kinase domain of mTOR, thereby activating mTOR kinase and downstream effector proteins (e.g. S6K and AKT). Moreover, the hydroxylation of P2341 strengthens mTOR stability and allows mTOR to accurately recognize its substrates such as S6K and AKT. The growth of lung adenocarcinoma cells overexpressing mTOR^P2341A^ is significantly reduced when compared with that of cells overexpressing mTOR^WT^. Interestingly, in vivo cell growth assays show that targeting P4HA2-mTOR significantly suppresses lung adenocarcinoma cell growth. In summary, our study reveals an undiscovered hydroxylation-regulatory mechanism by which P4HA2 directly activates mTOR kinase, providing insights for therapeutically targeting mTOR kinase-driven cancers.

## Introduction

Target of rapamycin (TOR), an evolutionarily conserved serine/threonine protein kinase that integrates a variety of stimuli including growth factors, nutrients, oxygen availability, and stress, functions as a central regulator of cell growth and metabolism by forming two distinct complexes: TORC1 and TORC2 [1–3]. Mammalian TORC1 (mTORC1) and mTORC2 exert their function through controlling several important kinases, such as S6 kinase (S6K) and AKT [4]. Dysregulation of mTOR signaling is commonly implicated in human diseases that include diabetes, neurodegeneration, and cancer [4, 5]. Arsham et al. previously reported that hypoxia resulted in the hypo-phosphorylation of mTOR and its targets including S6K and AKT, and hypoxia-induced inhibition of mTOR was dominant over mTOR activation via multiple environmental cues and occurred independently of HIF-1α [6], suggesting that mTOR activity is regulated by molecular oxygen (O_2_). However, little is known about the underlying mechanisms by which hypoxia negatively regulates mTOR activity in response to growth factors [7]. In addition, α-ketoglutarate (α-KG), a substrate required for activation of prolyl hydroxylase [8], indirectly activates mTORC1 by promoting GTP loading of RagB [9]. These findings indicate that O_2_ and α-KG are involved in mTOR kinase activity.

The mTOR was first identified in 1994 [10–12]. More recently, Hall’s research group comprehensively reviewed the literature regarding mTOR, and pointed out that much remains undetermined even though mTOR has been investigated for three decades [1]. For example, to date, the molecular function of mTOR phosphorylation is not defined, especially for mTOR activation. Figueiredo et al. suggested that phosphorylation at Ser2448 of mTOR is inadequately used as a measure of mTOR kinase activity in muscle metabolism, and recommend that evaluating mTOR activation should preferentially focus on downstream effector proteins (e.g. S6K) along mTOR protein-binding partners [13]. Therefore, it is still a puzzle for how mTOR itself is directly activated in cell growth and metabolism.

Here, we report that prolyl 4-hydroxylase alpha-2 (P4HA2), an O_2_ and α-KG-dependent enzyme [14], directly interacts with mTOR kinase and thereby activating mTOR signaling, and leading to the high-level phosphorylation of S6K-T389 (S6K^389^) and AKT-S473 (AKT^473^). Using mass spectrometry (MS)-based proteomic analysis in human embryonic kidney (HEK) 293T cells, we identified that P4HA2 hydroxylates one highly conserved proline 2341 (P2341) within a kinase domain of mTOR. Moreover, the hydroxylation of P2341 by P4HA2 is required for mTOR stability, whereas non-hydroxylated mTOR is degraded. P2341A (proline 2341 to alanine) mutant of mTOR caused low-level phosphorylation of S6K^389^ and AKT^473^, significantly reducing cell growth in A549 lung adenocarcinoma (LUAD) cells. P4HA2 knockdown and AZD-8055 (mTOR kinase inhibitor) synergistically suppressed LUAD cell growth. The combination of aspirin (potentially targeting P4HA2, [15]) and AZD-8055 had stronger inhibitory effects on LUAD cell growth. This provides a novel clue for therapeutically targeting mTOR kinase-driven cancers. Furthermore, structural analysis and cell-based experiments suggest that hydroxylation of P2341 endows mTOR with effective and accurate recognition of substrates such as S6K and AKT.

## Results

### P4HA2 directly interacts with mTOR kinase

**Fig. 1:**
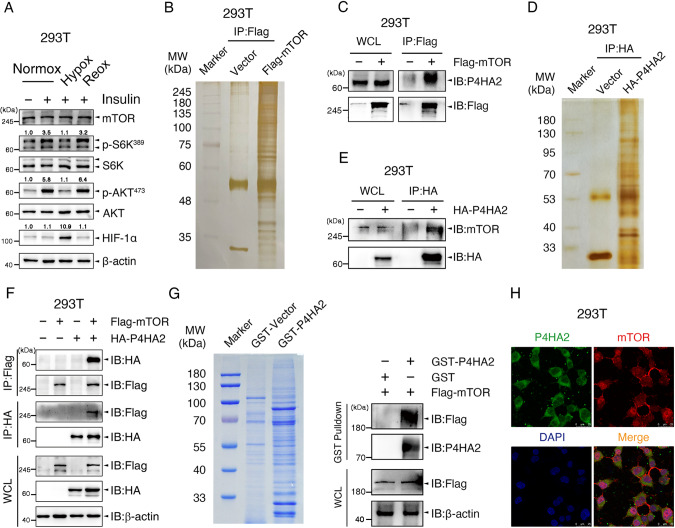
P4HA2 interacts with mTOR. **A** Serum-starved 293T cells were incubated in normoxia or hypoxia for 30 min, and then stimulated with 200 nM insulin for 45 min and reoxygenated for 1 h as indicated. Whole-cell lysates (WCL) were immunoblotted for mTOR, total or phosphorylated S6K, AKT, and HIF-1α. β-actin served as internal control. HIF-1α, Hypoxia-inducible factor-1 alpha. Densitometric quantification of the immunoblot bands was included in Fig. S1A. **B**, **C** WCL from 293T cells transfected with Flag-mTOR were immunoprecipitated with anti-Flag antibody, and bound proteins were eluted and subjected to silver staining (**B**) and MS-based proteomic analysis. The mTOR-binding endogenous P4HA2 was detected by immunoblot (**C**). IP, Immunoprecipitation; IB, Immunoblot. **D**, **E** WCL from 293T cells transfected with HA-P4HA2 were immunoprecipitated with anti-HA antibody, and bound proteins were eluted and subjected to silver staining (**D**) and MS-based proteomic analysis. The P4HA2-binding endogenous mTOR was detected with immunoblot (**E**). **F** WCL of 293T cells co-expressing Flag-mTOR and/or HA-P4HA2 were subjected to co-IP using anti-Flag or anti-HA antibodies. **G** Recombinant Glutathione S-transferase (GST)-P4HA2 pulls down Flag-mTOR in a cell-free system as described in STAR Methods. **H** 293T cells were co-incubated with a rabbit anti-P4HA2 antibody and a mouse anti-mTOR antibody and then stained with fluorescein isothiocyanate (FITC)-conjugated anti-rabbit IgG (green, for P4HA2) and Cy3-conjugated anti-mouse IgG (red, for mTOR). Cell nuclei were counterstained and visualized with DAPI. Scale bar: 10 μm.

**Fig. 2:**
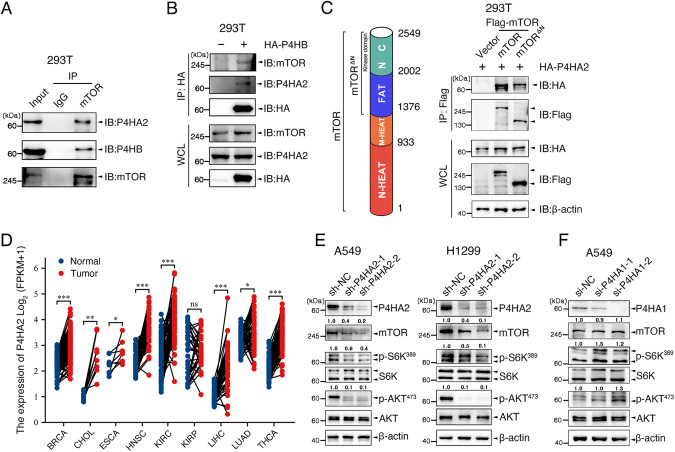
P4HA2 affects mTOR function. **A** Co-IP was conducted in 293T cells. Endogenous P4HA2 or P4HB were immunoprecipitated with anti-mTOR antibody. The binding of P4HA2 or P4HB to mTOR was determined by immunoblot. **B** Co-IP was performed in 293T cells overexpressing HA-P4HB. Endogenous mTOR or P4HA2 were immunoprecipitated with anti-HA antibody. The binding of mTOR or P4HA2 to P4HB was determined by immunoblot. **C** N-terminally truncated mTOR ((mTOR^ΔN^)) was shown in the schematic diagram of mTOR protein as indicated (left panel). Whole-cell lysates (WCL) of 293T cells transfected with Flag-mTOR or Flag-mTOR^ΔN^ and HA-P4HA2 were subjected to co-IP using anti-Flag antibody and immunoblotted with indicated antibodies (right panel). **D** Analysis of P4HA2 mRNA expression in pan-cancer tissues and normal tissues using TCGA database. BRCA breast invasive carcinoma, CHOL cholangio carcinoma, ESCA esophageal carcinoma, HNSC head and neck squamous cell carcinoma, KIRC kidney renal clear cell carcinoma, KIRP kidney renal papillary cell carcinoma, LIHC liver hepatocellular carcinoma, LUAD lung adenocarcinoma, THCA thyroid carcinoma, ns not significant; *P < 0.05, **P < 0.001 and ***P < 0.001 by paired Student’s *t*-test. **E** Immunoblot analysis of P4HA2, mTOR, total or phosphorylated S6K, and AKT in WCL from A549 and H1299 cells transfected with sh-P4HA2. β-actin served as internal control. Densitometric quantification of the immunoblot bands was included in Fig. S1B. **F** WCL of A549 cells transfected with si-P4HA1 were immunoblotted with antibodies against P4HA1, mTOR, total or phosphorylated S6K, and AKT. β-actin served as internal control. Densitometric quantification of the immunoblot bands was included in Fig. S1C.

In 293T cells treated with insulin, we found that the hypoxia-mediated dephosphorylation of S6K^389^ and AKT^473^ was rapidly reversed after 1 h of reoxygenation (Figs. 1A and S1A), supporting that O_2_ may regulate mTOR kinase activity. To identify candidate mTOR-interacting molecules, we performed an anti-Flag immunoprecipitation (IP) followed by MS-based proteomic analysis in 293T cells overexpressing Flag-tagged mTOR (Flag-mTOR) under normoxia condition. Among 634 potential mTOR-interacting proteins (Table S1A), prolyl 4-hydroxylase (P4H) subunit alpha-2 (P4HA2) was paid more attention (Fig. 1B) in view of the facts that a prolyl hydroxylase may bind to its cofactors, Fe^2+^ and α-KG, its substrate and O_2_ to transiently yield a prolyl hydroxylation reaction [14] and P4HA2 plays an essential role in oxygen sensing [16]. Then we confirmed whether mTOR interacts with P4HA2. In 293T cells transfected with Flag-mTOR, endogenous P4HA2 was co-immunoprecipitated with Flag-mTOR (Fig. 1B, C), and vice versa (Table S1B; Fig. 1D, E). Using co-IP experiments in 293T cells co-transfected with Flag-mTOR and/or HA-P4HA2, we found that P4HA2 and mTOR were successfully coprecipitated with mTOR and P4HA2, respectively (Fig. 1F). In a cell-free system, recombinant glutathione S-transferase (GST)-tagged P4HA2 pulled down Flag-mTOR in vitro (Fig. 1G), indicating a direct interaction between P4HA2 and mTOR. In support of this, immunofluorescence staining analyses showed that P4HA2 and mTOR were co-localized in the cytoplasm (Fig. 1H). More co-IP experiments showed that a small percentage of P4HA2 and P4HB, α- and β-subunits of P4H, interacted with mTOR in 293T cells (Fig. 2A, B), reflecting the dynamic nature of enzyme-substrate interactions. Furthermore, N-terminally truncated mTOR (mTOR^ΔN^) containing kinase domain (residues 1376-2549) was especially found to bind with P4HA2 (Fig. 2C). The interaction between endogenous P4HA2 and mTOR was further confirmed in LUAD cell line A549 cells (Fig. S2A).

Based on the evidence that dysregulation of mTOR signaling [4, 5] and upregulation of P4HA2 and mTOR expression in human cancers including LUAD (Figs. 2D and S2B), we subsequently focused on LUAD to investigate whether P4HA2 affects mTOR function. Interestingly, short hairpin RNA (shRNA)-mediated silencing of P4HA2 not only diminished mTOR expression but also inhibited S6K^389^ and AKT^473^ phosphorylation in LUAD cell lines A549 and H1299 (Figs. 2E and S1B), suggesting that P4HA2 positively regulates mTOR signaling. This is partially consistent with the findings in glioma, where P4HA2 silencing led to a reduction in phosphorylation levels of AKT [17]. Moreover, we found that shRNA-mediated silencing of P4HA2 inhibited cell growth in A549 and H1299 cells (Fig. S2C–F). In fact, P4HA1 and P4HA2 are two major isoforms of P4HA, which individually form α2β2 tetramers with P4HB and thereby contributing to P4HA enzyme activity in most cell types [18, 19]. However, siRNA-mediated knockdown of P4HA1 failed to restrain mTOR signaling in A549 cells (Figs. 2F and S1C). These findings suggest an important role of P4HA2 in mTOR signaling activation and LUAD cell growth.

### P4HA2 activates mTOR signaling and hydroxylates mTOR kinase domain at proline 2341

**Fig. 3:**
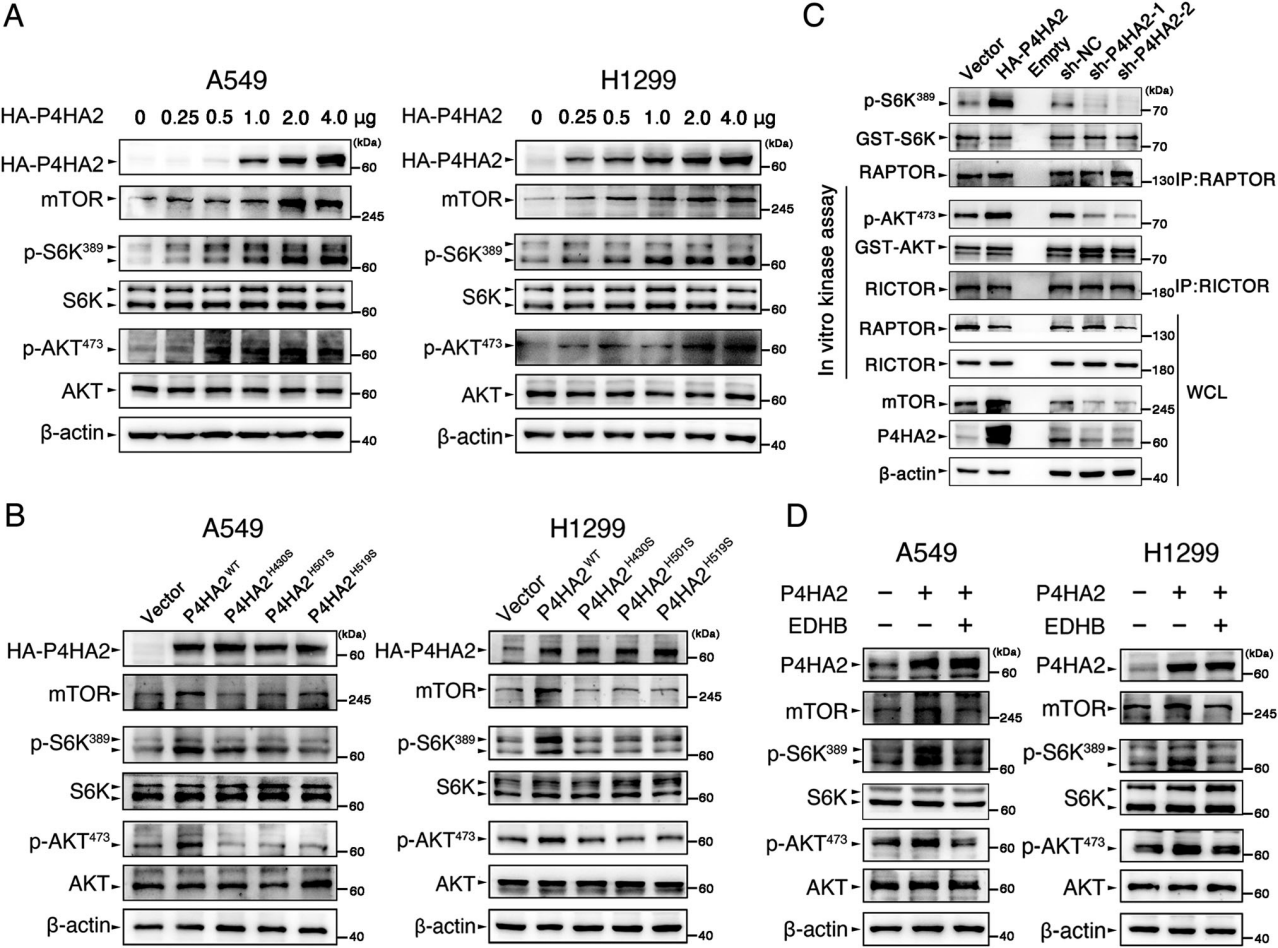
P4HA2 activates mTOR signaling. **A** Immunoblot analysis of HA-P4HA2, mTOR, total or phosphorylated S6K, and AKT in A549 and H1299 cells transfected with an increasing dose of HA-P4HA2. Densitometric quantification of the immunoblot bands was included in Fig. S1D. **B** A549 and H1299 cells were transfected with wild-type P4HA2 (HA-P4HA2^WT^) and hydroxylation-deficient P4HA2 mutants (HA-P4HA2^H430S^, HA-P4HA2^H501S^, and HA-P4HA2^H519S^) for 48 h, and WCL were immunoblotted for HA-P4HA2, mTOR, total or phosphorylated S6K and AKT. Densitometric quantification of the immunoblot bands was included in Fig. S1E. **C** Insulin (200 nM)-stimulated 293T cells with P4HA2 overexpression and sh-P4HA2 were immunoprecipitated with anti-RAPTOR or anti-RICTOR antibodies, and the immunoprecipitates were subjected to an assay of mTOR in vitro kinase activity by using recombinant S6K (50 ng) or AKT (50 ng) as mTORC1 and mTORC2 substrates. Densitometric quantification of the immunoblot bands was included in Fig. S1F. **D** P4HA2-overexpressing A549 and H1299 cells were treated with ethyl-3,4-dihydroxybenzoate (EDHB, 200 μM) for 24 h, and the immunoblot analysis was conducted as described in Fig. 2E. Densitometric quantification of the immunoblot bands was included in Fig. S1G.

Given that P4HA is metabolically regulated by α-KG during collagen hydroxylation [20] and α-KG associates with mTOR kinase activity [9], we hypothesized that P4HA2 activates mTOR via a hydroxylation regulation. To test this, we firstly monitored the changes in total mTOR abundance, and p-S6K^389^ and p-AKT^473^ levels in A549 and H1299 cells transfected with an increasing dose of P4HA2. Results of this experiment showed that the amount of mTOR, p-S6K^389^, and p-AKT^473^ elicited an increasingly dynamic response to P4HA2 (Figs. 3A and S1D). When compared with A549 and H1299 cells overexpressing wild-type P4HA2, the cells with three hydroxylation-deficient mutants of P4HA2 [21] presented lower levels of mTOR, p-S6K^389^ and p-AKT^473^ (Figs. 3B and S1E). S6K and AKT serve as substrates of mTOR kinase, and their phosphorylation status may directly reflect mTOR kinase activity. Thus, using an assay of mTOR in vitro kinase activity (The estimates of kinase activities were derived from the ratio of phosphorylated substrate to the total inactive substrate present in each reaction) [22], we found that P4HA2 overexpression and silencing respectively increased and reduced mTOR-catalyzed phosphorylation of S6K^389^ and AKT^473^ (Figs. 3C and S1F), further demonstrating the promotive effect of P4HA2 on mTOR activation. Moreover, ethyl-3,4-dihydroxybenzoate (EDHB), an α-KG mimic, was used as a P4HA2 enzymatic inhibitor [23] to treat P4HA2-overexpressing A549 and H1299 cells, resulting in attenuated levels of p-S6K^389^ and p-AKT^473^ (Figs. 3D and S1G). Moreover, EDHB inhibited the protein expression of mTOR and hydroxylation levels of total protein in A549 cells, without altering the expression of P4HA2 (Fig. S3). These results imply a hydroxylation role of P4HA2 for regulating mTOR signaling.

**Fig. 4:**
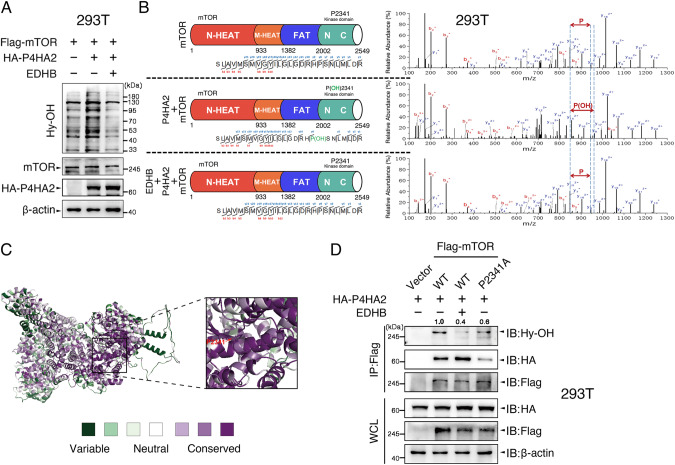
P4HA2 hydroxylates mTOR kinase domain at proline 2341. **A** 293T cells were co-transfected with Flag-mTOR and HA-P4HA2, and treated with EDHB (200 μM) as indicated. WCL were immunoblotted with antibodies against pan-prolyl hydroxylation (Hy)-OH, mTOR, HA, and β-actin as loading control. **B** 293T cells were transfected with Flag-mTOR, Flag-mTOR/HA-P4HA2 for 24 h, respectively. Flag-mTOR/HA-P4HA2-overexpressing cells were treated with EDHB (200 μM) for 24 h. WCL was harvested, and anti-Flag IP was performed to enrich mTOR protein followed by MS analysis. The fragmentation spectrum of mTOR peptide [_2123_SLAVMSMVGYILGLGDRH**P**(OH)SNLMLDR_2348_] carrying P2341 hydroxylation modification is shown with annotated with y-ions (blue) and b-ions (red). The y-ion series displays +16 Da mass shifts at the y^8^ ion (middle panel). P(OH) represents hydroxylated-proline. **C** Amino acid conservation of P2341 among different species was analyzed using the program ConSurf [27]. **D** 293T cells were co-transfected with HA-P4HA2 and wild-type Flag-mTOR or proline 2341 to alanine (P2341A) mutants of mTOR, and treated with EDHB as indicated. WCL were used for anti-Flag IP experiments and then immunoblotted with antibodies against pan-prolyl hydroxylation (Hy)-OH, HA, and Flag. Densitometric quantification of the immunoblot bands was included in Fig. S1H.

Inspired by our aforementioned findings, we speculate that mTOR can be hydroxylated by P4HA2. Thus, using 293T cells overexpressing Flag-mTOR, Flag-mTOR/HA-P4HA2, and Flag-mTOR/HA-P4HA2-overexpressing cells treated with EDHB, we observed that the hydroxylation levels of total protein were elevated in Flag-mTOR/HA-P4HA2-overexpressing cells and this increment was abolished by EDHB treatment (Fig. 4A). Next, we conducted an anti-Flag IP to capture and enrich mTOR protein, and the immunoprecipitates were subjected to MS analysis (Fig. 4B). Intriguingly, we identified a mTOR peptide sequence [_2123_SLAVMSMVGYILGLGDRH**P**^**#**^SNLMLDR_2348_] carrying P2341 hydroxylation modification, which exhibits +16 Da mass shifts at the y^8^ ion of the fragmentation spectrum (Fig. 4B, middle panel). The raw data of MS-based proteomics generated in Fig. 4B have been deposited into the ProteomeXchange Consortium via the iProX partner repository with the dataset identifier PXD041531. Therefore, MS analysis discovered P4HA2-catalyzed hydroxylation of the mTOR kinase domain at proline 2341, which positioned within the H-P-S motif. This is supported by the notion that P4HA2-induced hydroxylation sites are not limited to the X_(aa)_-P-G triplets [24–26]. Using the program ConSurf [27], we calculated that P2341 is highly conserved in the mTOR kinase domain (Fig. 4C), suggesting a crucial role of P2341 in controlling mTOR function. Moreover, P2341A (proline 2341 to alanine) mutant of mTOR (mTOR^P2341A^) significantly suppressed not only P4HA2-induced proline hydroxylation of mTOR but also the interaction between mTOR and P4HA2 (Figs. 4D and S1H), indicating that P4HA2 directly and specifically hydroxylates mTOR on P2341.

### P4HA2-catalyzed hydroxylation is required for mTOR stability

**Fig. 5:**
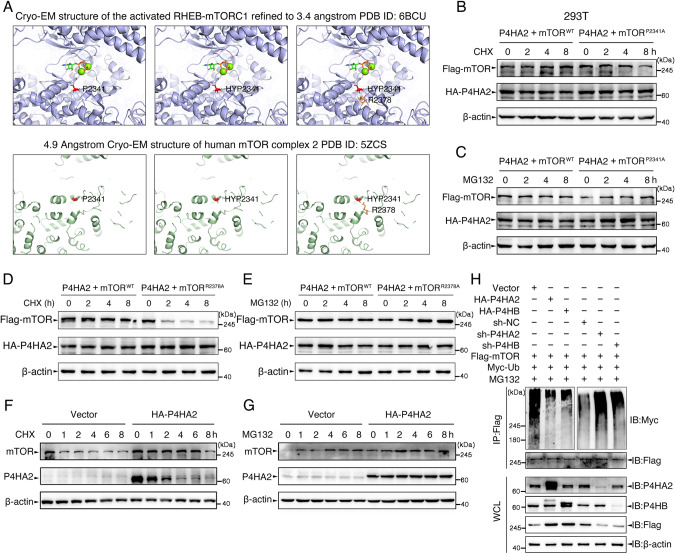
P4HA2-catalyzed hydroxylation is required for mTOR stability. **A** Crystallographic structure of mTORC1 (PDB:6BCU) and mTORC2 (PDB:5ZCS) showing the positions of P2341 of mTOR-catalyzed substrate binding site and R2378. HYP2341, hydroxylated P2341. **B**–**E** 293T cells co-transfected with Flag-mTOR^WT^ or Flag-mTOR^P2341A or R2378A^ and HA-P4HA2 were treated with CHX (50 μM) or MG132 (25 mM) for the indicated times, and immunoblotted with antibodies against Flag, HA and β-actin as loading control. CHX, protein biosynthesis inhibitor cycloheximide; MG132, proteasome inhibitor. **F**, **G** 293T cells overexpressing P4HA2 and empty vector were treated as above. Endogenous mTOR and P4HA2 expression were detected by immunoblot. **H** 293T cells were co-transfected with Flag-mTOR, Myc-Ubiquitin (Ub), HA-P4HA2 and HA-P4HB, or sh-P4HA2 and sh-P4HB and then treated with MG132 (25 mM) for 8 h as indicated, WCL were immunoprecipitated with anti-Flag antibodies and immunoblotted with the indicated antibodies.

The hydroxylation of proline residues in collagen strengthens the folding and stability of collagen [28, 29]. Qi et al. also linked increased Argonaute 2 (Ago2) stability to hydroxylation of Ago2 at proline 700 [30]. Coincidentally, in this study, the hydroxylated P2341 (HYP2341) of mTOR may be negatively charged at physiological pH (~6.50) and evoke electrostatic forces because pH 6.30 is the isoelectric point for proline and pH 5.83 for HYP. Therefore, HYP2341 can electrostatically interact with positively charged arginine 2378 (R2378), and this interplay is supported by using Cryo-EM structure analyses in mTORC1 and mTORC2 (Fig. 5A). These data encouraged us to explore whether HYP2341 regulates mTOR stability by interacting with R2378. As expected, mTOR^P2341A^ led to the destabilization of mTOR in protein biosynthesis inhibitor cycloheximide (CHX)-treated 293T cells overexpressing P4HA2 (Fig. 5B), but this effect was blocked by the proteasome inhibitor MG132 (Fig. 5C). R2378A (arginine 2378 to alanine) mutant of mTOR (mTOR^R2378A^) resulted in the same effects as mTOR^P2341A^ (Fig. 5D, E). Moreover, the stabilization of endogenous mTOR by P4HA2 overexpression (Fig. 5F) was further enhanced by MG132 (Fig. 5G), suggesting that non-hydroxylated mTOR could be degraded via a ubiquitin-mediated proteasome-dependent mechanism. Thus, we examined the ubiquitylation of mTOR in the presence or absence of P4HA2 and P4HB. Co-IP experiments revealed that P4HA2 or P4HB overexpression inhibited mTOR polyubiquitylation in 293T cells, whereas P4HA2 or P4HB silencing had the opposite effect (Fig. 5H). Collectively, these results provide a notion that the hydroxylation of P2341 by P4HA2 is required for mTOR stability.

Considering the previous findings that mTOR and suppressor of morphogenesis in genitalia 1 (SMG1) belong to the phosphatidylinositol kinase-related kinase family [31, 32] and mTOR-CDK2 superposition reveals a coincidence of mTOR H2340 and CDK2 K129 side chains [33], we focused on insights into the structural position of mTOR P2341 site and found that HYP2341 is just located in the catalytic pocket. This is evidenced by the mTOR-SMG1-CDK2 superposition (Fig. S4A–E), suggesting that hydroxylation of P2341 is a critical event causing mTOR activation.

### Hydroxylation of mTOR P2341 affects substrate phosphorylation and recognition of mTOR

**Fig. 6:**
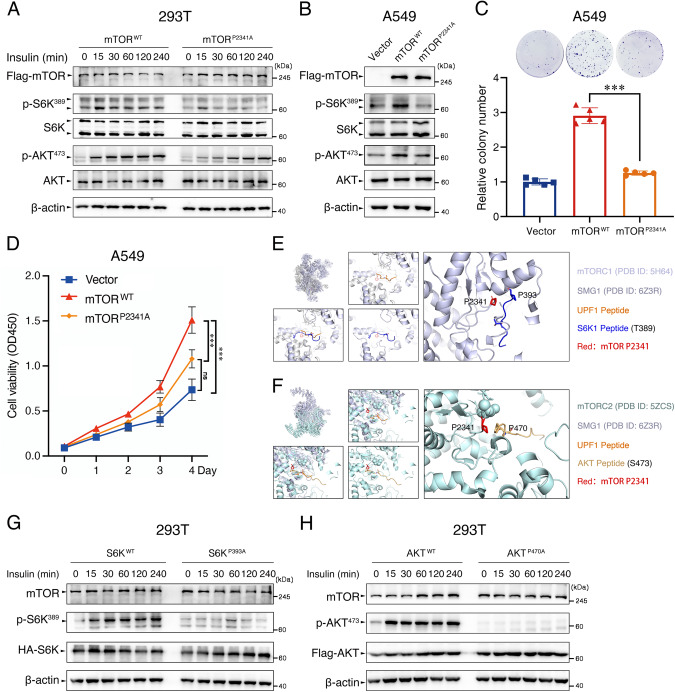
Hydroxylation of mTOR P2341 affects substrate phosphorylation and recognition of mTOR. **A** Serum-starved 293T cells transfected with Flag-mTOR^WT^ or Flag-mTOR^P2341A^ were stimulated with insulin (200 nM) for the indicated times. The WCL were immunoblotted with antibodies against Flag, total or phosphorylated S6K, and AKT. **B** Immunoblot analysis of mTOR, total or phosphorylated S6K, and AKT in A549 cells with Flag-mTOR^WT^ or Flag-mTOR^P2341A^. Densitometric quantification of the immunoblot bands was included in Fig. S1I. **C**, **D** Colony formation and CCK8 assays were performed in the above-mentioned A549 cells (**B**). Data are shown as the mean ± SD (*n* = 5). ns not significant; ***P < 0.001 by unpaired Student’s *t*-test. **E**, **F** Superimposition of the substrate binding grooves of SMG1 (PDB: 6Z3R) and mTORC1 (PDB: 5H64) or mTORC2 (PDB: 5ZCS) was visualized using the PyMol software. S6K1 substrate peptide containing P393 near T389 or AKT substrate peptide containing P470 near S473 were overlaid into the catalytic pockets of mTORC1 or mTORC2, respectively. This overlay yields the contact of mTOR P2341 with P393 embodied in the S6K1 substrate peptide, or the contact of mTOR P2341 with P470 embodied in the AKT substrate peptide. **G**, **H** 293T cells transfected with HA-S6K^WT^ and HA-S6K^P393A^, or Flag-AKT^WT^ and Flag-AKT^P470A^ were serum-starved for 24 h and stimulated with insulin (200 nM). The WCL were immunoblotted with antibodies against mTOR, HA or Flag, phosphorylated S6K^389^, and AKT^473^.

Next, we determined the role of mTOR P2341 hydroxylation in mTOR substrate phosphorylation and cell growth. Upon insulin stimulation, 293T cells overexpressing a hydroxylation-deficient mutant of mTOR (mTOR^P2341A^) exhibited significantly reduced activation of mTOR, showing lower levels of p-S6K^389^ and p-AKT^473^ (Fig. 6A). Even if P4HA2 was overexpressed in 293T cells, the incorporation of mTOR^R2378A^ mutant partially decreased mTOR kinase activity (Fig. S4F). Interestingly, we did not find any cancer-associated mTOR mutations at P2341 and R2378 (Table S2A) using the cBio Cancer Genomics Portal (http://cbioportal.org) [34], inspiring us to investigate the significance of mTOR P2341 hydroxylation modification for cancer progression. Overexpression of mTOR^P2341A^ in A549 cells caused a significant reduction in phosphorylation of mTOR-activated substrates S6K or AKT (Figs. 6B and S1I), and thereby attenuating tumor cell growth in vitro (Fig. 6C, D) and in vivo (Fig. S5A–E).

Hydroxyproline residues have a key role in stabilizing collagen [28, 29]. The pyrrolidine ring of HYP makes conformationally contacts with the pyrrolidine ring in the triple-helical structure of collagen (Fig. S6A). Therefore, it is likely that HYP2341 contributes to substrate recognition of mTOR. Overlapping the structure of mTORC1 or mTORC2 catalytic pocket with SMG1, we predicted that like collagen, mTOR recognizes substrates in the catalytic pocket composed of HYP-proline contact structure (Figs. 6E, F, and S6B–G). Thus, we tested whether the contact is required for the recognition of substrate and affects insulin-stimulated mTOR kinase activity. As a result, in 293T cells treated with insulin, S6K^P393A^ and AKT^P470A^ mutants abolished mTORC1-catalyzed S6K-T389 phosphorylation and mTORC2-catalyzed AKT-S473 phosphorylation, respectively. (Fig. 6G, H). No cancer-associated mutations were found at S6K-P393 and AKT-P470 (Table S2B, C), indicating their significance in substrate recognition of mTOR.

### Targeting P4HA2-mTOR suppresses LUAD cell growth

**Fig. 7:**
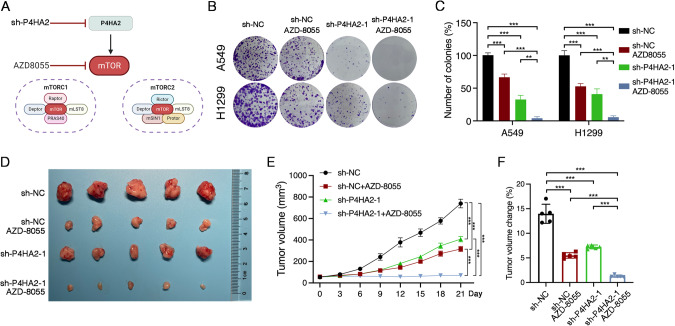
Targeting P4HA2-mTOR inhibits LUAD cell growth. **A** Schematic illustration of targeting P4HA2-mTOR through P4HA2 knockdown and mTOR kinase inhibitor AZD-8055. **B**, **C** A549 and H1299 cells with P4HA2 knockdown were treated with AZD-8055 (1 nM) for 7 days and subjected to colony formation assays. Data are shown as the mean ± SD (*n* = 5). **P < 0.01, ***P < 0.001 by unpaired Student’s *t*-test. **D** Tumor size analysis of the surgically excised tumors from individual BALB/c nude mice. Mice bearing sh-NC or sh-P4HA2-1 A549 xenograft tumors (5 mice per group) were randomized to four groups [sh-NC, sh-NC+AZD-8055 (10 mg/kg), sh-P4HA2-1, and sh-P4HA2-1+AZD-8055]. **E**, **F** Tumor volume changes (**E**) of the xenografts following treatment as mentioned above. Tumor size was measured using calipers every 3 days. Dot plot (**F**) showing changes in tumor volume after 21 days of treatment. Data are shown as the mean ± SD (*n* = 5); ***P < 0.001 by unpaired Student’s *t*-test.

Given our findings that P4HA2 activates mTOR, we hypothesized that P4HA2 knockdown and mTOR kinase inhibitor AZD-8055 exhibit stronger antitumor efficacy by inhibiting LUAD cell growth (Fig. 7A). To test this, we firstly used AZD-8055 to treat A549 and H1299 cells with P4HA2 knockdown and found that AZD-8055 led to a significant reduction of cell growth in A549 and H1299 cells with P4HA2 knockdown (Fig. 7B, C). Next, A549 cells with P4HA2 knockdown and control cells were subcutaneously implanted into BALB/c nude mice. When tumor size reached 50–70 mm^3^, we injected AZD-8055 into the mice every 3 days. Tumor-bearing mice were euthanized 21 days after drug treatment, and the excised tumors showed remarkably smaller in mice inoculated with AZD-8055 (Fig. 7D–F), indicating that P4HA2 knockdown and AZD-8055 synergistically inhibited LUAD cell growth. Because there is a published report suggesting that aspirin targets P4HA2 to decrease collagen deposition [15], we assessed the effect of a combination of aspirin and AZD-8055 on LUAD cell growth (Fig. S7A). As a result, we found that aspirin and AZD-8055 significantly inhibited the growth of A549 and H1299 cells compared with single-agent treatment (Fig. S7B, C). In tumor-bearing mice, we observed that the combined treatment groups with aspirin and AZD-8055 significantly suppressed tumor growth compared with single-agent treatment groups (Fig. S7D–F). Taken together, our results suggest that targeting P4HA2-mTOR may be a potential therapeutic strategy for LUAD.

## Discussion

The mTOR signaling plays a central role in the regulation of cellular translation, cell growth, and energy metabolism [2, 3], whose dysregulation is frequently implicated in human diseases including cancer [4, 5, 35]. Despite three decades of active research in mTOR kinase, much remains to be determined [1].

Hypoxia rapidly and reversibly triggers hypo-phosphorylation of mTOR and its substrates including S6K and AKT [6], suggesting the involvement of O_2_ regulation in mTOR phosphorylation and activation. However, the effect of mTOR phosphorylation has not been yet known [1] after proposing that the phosphorylation of mTOR does not indicate its activation [35]. In this study, using HEK 293T and LUAD cell lines, we discover an important role of P4HA2-induced prolyl hydroxylation in mTOR activation. In support of our findings, P4HA requires O_2_ and α-KG to generate a prolyl hydroxylation reaction [14], α-KG may regulate P4HA in the hydroxylation of collagens and other proteins with collagen-like sequences [20] and indirectly correlates with mTOR kinase activity [9].

Importantly, we identified that a crucial hydroxylation site P2341 within mTOR kinase is required for mTOR activation and stability, and P2341A (proline 2341 to alanine) mutant not only markedly inhibits the phosphorylation of mTOR-catalyzed substrates S6K or AKT but also LUAD cell growth. Given that more than 100 mutation sites rather than P2341 within the mTOR kinase domain have been found in many types of cancers, including LUAD (http://cbioportal.org) [34] and P4HA2 is up-regulated in human cancers (TCGA database), we believe that P2341 is favorable for mTOR activation and tumor cell growth. These data provide insights for therapeutically targeting mTOR kinase-driven cancers. Upregulation of mTOR signaling can promote tumor growth and progression through diverse mechanisms [36]. Thus, mTOR signaling has become an attractive target for cancer therapy. However, mTOR inhibitors such as rapamycin and its derivatives have not shown great efficacy [37], suggesting that new ways of inhibiting mTOR signaling are important. In this study, we demonstrate that P4HA2 inhibition leads to the downregulation of mTOR and decreased activation of downstream effectors like AKT. The results of in vitro and in vivo experiments show greater antitumor ability of targeting P4HA2-mTOR, indicating the importance of P4HA2-induced mTOR activation in LUAD and its significance as an emerging therapeutic target for treating cancer. In fact, strengthening protein stability by P4HA is not surprising because the hydroxylation of Ago2 at proline 700 has been proved to improve Ago2 stability [30]. Nevertheless, Qi et al. did not reveal the mechanisms behind hydroxylation-promoted Ago2 protein stability. Our study shows that non-hydroxylated mTOR can be degraded via a ubiquitin-mediated proteasome-dependent mechanism. Notably, this is different from the previous findings that non-hydroxylated HIF-1α avoids degradation by E3 ubiquitin ligase von Hippel-Lindau (VHL), a tumor suppressor protein [38, 39].

Until now, another key question of how distinct substrates are recognized and recruited to mTORC1/2 is unanswered [1]. Intriguingly, using a structural superposition of the substrate binding grooves of SMG1 and mTORC1/2, we predicted that a particular contact between the hydroxylated P2341 and the prolines (e.g. S6K-P393 and AKT-P470) embodied in mTOR substrate peptides may contribute to substrate recognition. In our example, S6K^P393A^ and AKT^P470A^ substrate mutants respectively abrogated mTORC1-catalyzed S6K-T389 phosphorylation and mTORC2-catalyzed AKT-S473 phosphorylation, presenting a specific conformational adaptation in mTOR kinase-substrate interaction.

An important limitation of this study is that other protein kinases, except mTOR, are also probably activated through P4HA2-mediated prolyl hydroxylation. To overcome this limitation, we expect that other candidate P4HA2-interacting protein kinases such as Aurora kinase A and Cyclin-dependent kinase 13 available from our MS-based analysis can be validated to activate in a proline-hydroxylation manner. Although we found a ubiquitin-mediated proteasome-dependent mechanism required for non-hydroxylated mTOR degradation, specific E3-ubiquitin ligases remain unclear. Additional experiments are needed to address this limitation regarding E3-ubiquitin ligases. Another limitation of our study is that 4E-BP1 or PKC substrate peptides recognition of mTOR kinase was not further confirmed by cell-based experiments, albeit predicting their reciprocal contact using the structural superposition. Although our study reveals the important role of P4HA2-induced prolyl hydroxylation in mTOR activation, there is no pharmacological agents targeting P4HA2 to effectively inhibit its enzyme activity [40]. Therefore, further researches regarding the development of P4HA2 inhibitors are needed.

Taken together, our experiments discover a hydroxylation-regulatory mechanism that activates and stabilizes mTOR kinase, and provide insights for therapeutically targeting mTOR kinase-driven cancers.

## Materials and methods

### Cell culture

Human embryonic kidney (HEK) 293T cell line, and lung adenocarcinoma (LUAD) cell lines A549 and H1299 were obtained from the Cell Bank of Chinese Academy of Sciences. All the cell lines were authenticated with STR profiling and checked free of mycoplasma contamination by PCR. HEK 293T cells were cultured in Dulbecco’s Modified Eagle medium with high glucose (DMEM, Thermo Scientific, 21013024) and 10% fetal bovine serum (FBS). A549 and H1299 cells were cultured in RPMI-1640 (Corning, 10-040-CV) with 10% FBS. Hypoxia treatment was made by culturing cells in a hypoxic chamber containing 1% oxygen (Coy Laboratory Products). Cells were serum-starved in DMEM for 24 h followed by stimulation with 200 nM insulin (Beyotime, P3376) for evaluating mTOR activation levels.

### Construction of vectors and transfections

The full-length coding sequence (CDS) of mTOR (GenBank Accession number: NM_001386500.1), P4HA2 (NM_001017974.2), S6K (NM_001272042.2), and AKT (NM_001014431.2) were amplified from 293T cell cDNA library and then subcloned into pcDNA3.1-Flag/HA vectors with endonucleases *Nhe*I/*Age*I (New England Biolabs, NEB; R0131L/R0552L) to generate pcDNA3.1-mTOR-Flag, pcDNA3.1-P4HA2-HA, pcDNA3.1-S6K-HA, and pcDNA3.1-AKT-Flag transient expression vectors. During the construction of above-mentioned vectors, the CDS of GFP was excised with *Nhe*I*/Age*I endonucleases and replaced with the CDS of the interested gene. The CDS of mTOR was subcloned into pCDH-Flag with endonucleases *Xba*I*/EcoR*I to yield a pCDH-mTOR-Flag stable expression vector. The mutant constructs containing mTOR^P2341A or R2378A^, P4HA2^H430S, H501S or H519S^, S6K^P393A^, and AKT^P470A^ were generated using a PCR-based site-directed mutagenesis and ClonExpress Ultra One Step Cloning Kit (C115-01, Vazyme). The pcDNA3.1-mTOR-Flag was used as a DNA template to yield the constructs containing N-terminally truncated mTOR (mTOR^ΔN^, residues 1376-2549). Primers used for plasmid construction were listed in Table S3. All plasmids were validated with Sanger sequencing (Tsingke). Next, HEK 293T, A549, or H1299 cells were transiently transfected with above-constructed vectors using Lipofectamine 3000 (Thermo Fischer, L3000015) (1 μg DNA: 2 μl Lipofectamine).

### Establishment of stable cell lines

Two shRNAs targeting P4HA2 (sh-P4HA2-1 and sh-P4HA2-2) and negative control shRNA (sh-NC) were synthesized by Tsingke (Table S3) and then subcloned into a lentiviral vector pLKO.1-puro (GENEWIZ) with endonucleases *Age*I/*Eco*RI (NEB, R0552L/R0101L) to create pLKO.1-sh-P4HA2-1/2 and pLKO.1-sh-NC. Subsequently, the aforementioned constructs, pCDH-mTOR^WT^-Flag, pCDH-mTOR^P2341A^-Flag, and pCDH-Flag vectors were respectively co-transfected with packaging plasmids psPAX2 and pMD2.G (RIBOBIO) into HEK 293T cells using Lipofectamine 3000. Packaged lentiviruses were collected and used to infect the selected cells for 72 h. Finally, stable cells were selected with 0.2 μg/ml puromycin (Solarbio, P8230).

### RNA interference

Two short interfering RNAs (siRNAs) targeting P4HA1 (si-P4HA1-1 and si-P4HA1-2) were designed and synthesized (GenePharma). A scrambled siRNA served as negative control (si-NC). The sequences of siRNAs are listed in Table S3. Cells were transiently transfected with 100 pmol of siRNAs using Lipofectamine 3000. At 48 h post-transfection, cells were harvested for further experiments.

### Immunoblot analysis

Immunoblot analysis was conducted as previously described by us [41]. The detailed protocol was described in the Supplementary Experimental Procedures. Each immunoblotting experiment was performed in at least three independent biological repeats.

### Co-immunoprecipitation (Co-IP)

Co-IP was performed as described previously [41] with some modifications. Briefly, cells were grown on 10-cm dishes and lysed with ice-cold IP buffer (50 mM Tris-HCl (pH 7.5), 150 mM NaCl, 0.5% NP-40, 10% glycerol) for 30 min. Cell lysates were incubated with magnetic beads carrying antibodies against Flag or HA (Bimake, B26102 or B26202), or incubated with the appropriate antibodies coupled to protein A/G agarose beads (Thermo Fisher Scientific, 88802) for 3 h at 4 °C. Lysates from cells expressing Flag/HA-GFP vectors served as negative control. Subsequently, the immunoprecipitated beads were washed with IP buffer. Immunoprecipitates and input were subjected to immunoblot or MS-based analysis.

### Protein silver-staining assay

The anti-Flag or anti-HA Co-IP products from HEK 293T cells transfected with Flag-mTOR or HA-P4HA2 were resolved on SDS-PAGE gel and then identified with Silver Staining Kit (CWBIO, CW2012). The protocol was performed as previously described [41].

### Mass spectrometry assay

The aforementioned IP protein mixture was eluted from beads with 8 M urea, and incubated with 10 mM DTT at 60 °C for 30 min, alkylated with 55 mM iodoacetamide (IAM) in the dark at room temperature for 45 min, and digested with trypsin at 37 °C overnight. The resulting tryptic peptides were purified by buffer A [0.2% trifluoroacetic acid (TFA)] and buffer B (80% acetonitrile and 0.2% TFA) using a C18 column (Millipore, ZTC18S096). Subsequently, the purified samples were dissolved in solvent A (0.1% formic acid), directly loaded onto an analytical column (15-cm length, 75-μm i.d.), and separated with the liquid chromatography gradient [0–5 min, 5–10% solvent B (0.1% formic acid and 80% acetonitrile); 5–89 min, 10–28% solvent B; 89–109 min, 28–38% solvent B; 109–114 min, 38–100% solvent B; 114–120 min, 100–100% solvent B], with a constant flow rate of 300 nL/min on an EASY-nLC 1200 UPLC system (Thermo Fisher Scientific). Eluted peptides were analyzed on an Orbitrap Fusion Lumos mass spectrometer (Thermo Fisher Scientific) in the positive-ion mode. The electrospray voltage applied was 2.0 kV. The scan range was from 350 to 1800 m/z for full-scan MS spectra. The resolutions of the MS and MS/MS scans detected in the Orbitrap were 60,000 and 17,500, respectively. The MS/MS normalized collision energy and dynamic exclusion time were individually set as 30% and 20 s, and automatic gain control was set at 5e4.

The MS/MS spectral data were searched against the human protein database from the UniProt database (https://www.uniprot.org) using the Proteome Discoverer 2.0 software (Thermo Scientific). Software parameters were set as follows: carbamidomethyl on cysteine (Cys) for fixed modification, oxidation on proline (Pro) for variable modification, a maximum of 2 trypsin missed-cleavage, mass tolerances of 10 ppm for precursor ions and 0.02 Da for fragment ions, 1% false discovery rate. The raw data of MS-based proteomics have been deposited into the ProteomeXchange Consortium via the iProX partner repository with the dataset identifier PXD041531.

### Immunofluorescence staining

The detailed protocol was described in the Supplementary Experimental Procedures.

### Expression and purification of GST fusion proteins

P4HA2, S6K, and AKT CDSs were amplified with the corresponding PCR primers (Table S3) and subcloned into pGEX-4T-2 vector with N-terminal GST-tag using endonucleases *Bam*HI/*Not*I (NEB, R0136L/R0189L) to yield pGEX-GST-P4HA2, pGEX-GST-S6K, and pGEX-GST-AKT expression vectors. *E. coli* strain BL21 cells were transformed with above-constructed vectors or pGEX-4T-2 (empty vector) and cultured at 37 °C overnight, and then induced with 0.1 mM isopropyl β-D-1-thiogalactopyranoside (IPTG) at 16 °C with vigorous shaking for 12 h. Cells were collected and sonicated on ice, and incubated with glutathione-Sepharose beads (Sangon Biotech, C600327). Purified GST-P4HA2 or GST proteins coupled to the beads were used for GST pull-down analysis, while purified and eluted GST-S6K and GST-AKT proteins were prepared for assay of mTOR in vitro kinase activity.

### GST pull-down

Purified GST-P4HA2 or GST proteins coupled to glutathione-Sepharose beads (Sangon Biotech, C600327) were mixed with lysates of HEK 293T cells overexpressing Flag-mTOR. The mixture was incubated by rotating at 4 °C overnight. After washing for three times, bound proteins were separated on SDS-PAGE gel and visualized with Coomassie Blue staining and immunoblot.

### Assay of mTOR in vitro kinase activity

An assay of mTOR in vitro kinase activity was done as previously described [22] with some modifications. Insulin (200 nM)-stimulated HEK 293T cells were lysed with the buffer (20 mM Tris, pH 7.5, 150 mM NaCl, 1× protease inhibitor cocktail, 1.0 mM 2-mercaptoethanol, 1.0% Triton, 10% Glycerol and 1.0 mM MgCl_2_). The lysates were immunoprecipitated with anti-RAPTOR and anti-RICTOR antibodies (0.4 μg for both). RAPTOR or RICTOR immunoprecipitates were washed in kinase buffer (25 mM HEPES pH 7.4, 20 mM KCl, and 10 mM MgCl_2_). Then, the complex mTORC1 (RAPTOR) or mTORC2 (RICTOR) was incubated with mTOR kinase buffer (25 mM HEPES, 50 mM KCl, 10 mM MgCl_2_, and 500 μM ATP) with bacterially purified inactive 50 ng GST-S6K (for mTORC1 complex) or 50 ng GST-AKT (for mTORC2 complex) substrate at 37 °C. After 30 min, the kinase-catalyzed reactions were stopped. Finally, the reaction mixture was subjected to immunoblot analysis with antibodies against p-S6K^389^ or p-AKT^473^. The mTOR kinase activities were assessed by the ratio of phosphorylated substrate to total substrate in each reaction.

### Identification of prolyl hydroxylation site of mTOR

HEK 293T cells were transfected with Flag-mTOR, Flag-mTOR/HA-P4HA2 for 24 h, respectively. Flag-mTOR/HA-P4HA2-overexpressing cells were treated with EDHB (200 μM) for 24 h. Forty-eight hours later, the whole-cell lysates from above-treated cells were collected and incubated with anti-Flag M2 Affinity Gel beads (Sigma-Aldrich, A2220) at 4 °C for 5 h. Then the Flag beads were washed with cold lysis buffer, and the IP protein mixture was eluted from beads with 8 M urea, followed by incubation with 10 mM DTT (final concentration) for 30 min, IAM solution at room temperature for 45 min in the dark. Next, 50 mM DTT solution was added to stop alkylation. Eluates containing mTOR protein were prepared for protease digestion (trypsin) and desalted with C18 Ziptip (Millipore, ZTC18S096). The pooled supernatants were vacuum concentrated to dryness and subjected to mass spectrometry analysis with Orbitrap Fusion Lumos (Thermo Scientific). The prolyl hydroxylated peptide spectrum (15.99491@Pro) matches for mTOR were obtained after Proteome Discoverer 2.0 database (Thermo Scientific) search.

### Colony formation and CCK8 assays

The detailed protocol was described in the Supplementary Experimental Procedures.

### Structural superimposition

A human SMG1 kinase complex bound to a UPF1 phosphorylation site was applied as the structure model. Superimposition of the substrate binding grooves of SMG1 with a UPF1 substrate peptide (PDB ID:6Z3R) and mTORC1 (PDB: 5H64) or mTORC2 (PDB: 5ZCS) was visualized using the PyMol software (Schrodinger LLC). The mTOR phosphorylation substrates were superimposed with the UPF1 substrate peptide for mimicking the binding of mTOR to its substrate peptides.

### In vivo LUAD cell growth assays

The Animal Ethics Committee of Soochow University has approved all the animal studies. BALB/c nude mice were purchased from the Laboratory Animal Center of Soochow University, maintained and treated under specific pathogen-free conditions.

Fifteen 4-week-old female BALB/c nude mice were randomly divided into three groups, including the control vector group, mTOR^WT^-overexpressing group, and mTOR^P2341A^-overexpressing group (5 mice per group). mTOR^WT^-overexpressing, mTOR^P2341A^-overexpressing, and control A549 cells were subcutaneously implanted into the right flank of mice (4 × 10^6^ cells/mouse). When tumors reached 50–70 mm^3^, tumors were measured with a caliper every 3 days to determine tumor volume. At the experimental endpoint, tumors were harvested and fixed with 4% PFA for paraffin-embedded section.

Twenty 4-week-old female BALB/c nude mice were randomly grouped and subcutaneously injected with A549 cells stable expressing sh-P4HA2-1 or sh-NC in the right flanks (4 × 10^6^ cells/mouse). Subsequently, the mice were randomly divided into four groups (5 mice per group): (i), sh-NC; (ii), sh-NC and AZD-8055 (10 mg/kg); (iii), sh-P4HA2-1; (iv), sh-P4HA2-1 and AZD-8055. When tumors reached 50–70 mm^3^, 200 μL AZD-8055 or DMSO was injected into the tail vein of mice every 3 days. Tumors were measured with a caliper every 3 days to analyze tumor growth. Tumors were harvested at the experimental endpoint.

Sixteen 4-week-old female BALB/c nude mice were subcutaneously injected with A549 cells (4 × 10^6^ cells/mouse) into the right flank. Once tumors became palpable, 7 days post-implantation, mice bearing A549 xenograft tumors were randomized to four groups (4 mice per group) and subjected to the following treatments for 21 days: (i) 200 μL DMSO (vehicle control), (ii) 200 μL aspirin (100 mg/kg), (iii) 200 μL AZD-8055 (10 mg/kg), and (iv) combined aspirin with AZD-8055. Drugs were injected into the tail vein of mice every 3 days. Tumor growth was monitored by caliper measurements.

Tumor volume was calculated using the formula: tumor volume (mm^3^) = *D* × *d*^2^/2, where *D* and *d* respectively represent the longest and the shortest diameters of the tumor. The cohorts of mice were sacrificed when the biggest tumors of the respective controls were approximately 1000 mm^3^.

### Immunohistochemistry (IHC) staining

The detailed protocol was described in the Supplementary Experimental Procedures.

### Statistical analysis

The difference between two groups was compared by paired or unpaired Student’s *t*-test. Data were expressed as mean ± SD. *P* values less than 0.05 were considered statistically significant. ImageJ software was used for quantifying protein levels from immunoblots. Statistical analyses were performed using GraphPad Prism 7.01 software (GraphPad).

### Supplementary information

Supplementary Experimental Procedures

Supplementary Figure Legends

Figure S1

Figure S2

Figure S3

Figure S4

Figure S5

Figure S6

Figure S7

Table S1 (Excel Table). Mass spectrometry-based proteomic analysis for identifying potential mTOR- and P4HA2-interacting proteins

Table S2 (Excel Table). List of mTOR kinase domain, S6K and AKT mutations found in human cancer patient samples

Table S3 (Excel Table). Primers used for plasmid construction and sequences of siRNAs and shRNAs

## Data Availability

The cBio Cancer Genomics Portal (http://cbioportal.org) was used to identify any cancer-associated mutations of mTOR, S6K, and AKT. The raw data of MS-based proteomics generated in Table S1 and Fig. 4B have been deposited into the ProteomeXchange Consortium via the iProX partner repository with the dataset identifier PXD041531.
