# Supplementary material for: P4HA2 activates mTOR via hydroxylation and targeting P4HA2-mTOR inhibits lung adenocarcinoma cell growth
Source: Oncogene. 2024 Apr 23;43(24):1813–23. doi: 10.1038/s41388-024-03032-1 (PMC11164680; doi:10.1038/s41388-024-03032-1)
Supplement: Supplementary file 1 — Supplementary Experimental Procedures [file 41388_2024_3032_MOESM1_ESM.docx]

**Supplementary Experimental Procedures**

**Immunoblot analysis**

Immunoblot analysis was conducted as previously described by us [[1](#_ENREF_1)]. Briefly, cells were collected and lysed in ice-cold RIPA buffer (Thermo Fisher Scientific, 89900) supplemented with protease inhibitor cocktail (Sangon Biotech, C600387) and phosphatase inhibitor cocktail (Sangon Biotech, C500017). Lysates were cleared by centrifugation at 14,000 g for 15 min at 4°C and total protein was quantitated using BCA Protein Quantification Kit (Yeasen Biotechnology, 20201ES76). Equal amounts of whole cell lysates were subjected to SDS-PAGE gel electrophoresis and transferred onto nitrocellulose membrane (Merck, HATF00010) followed by immunoblot with indicated antibodies. Eventually, proteins were detected by ECL chemiluminescence kit (Fdbio science, FD8020). Each immunoblotting experiment was performed in at least three independent biological repeats.

**Immunofluorescence staining**

Cells were seeded on coverslips in a 24-well culture plate. Twenty-four hours later, cells were washed with PBS buffer, fixed with 4% paraformaldehyde for 30 min, permeabilized with 0.2% Triton X-100, and then blocked with 5% BSA for 1 h. Subsequently, cells were incubated with rabbit anti-P4HA2 (Proteintech, 13759-1-AP) and mouse anti-mTOR (Santa Cruz, sc-51764) antibodies (1:500 dilution for both) at 4°C overnight. The following day, cells were washed with PBST buffer and incubated with FITC-conjugated anti-rabbit secondary antibody (Beyotime, A0562) and Cy3-conjugated anti-mouse secondary antibody (Beyotime, A5021) for 2 h in dark. Nuclei were counterstained with 4’,6-diamidino-2-phenylindole (DAPI). Finally, protein localization was observed and photographed using a confocal laser scanning microscope (Leica SP8), and images were digitally merged for co-localization examination. Immunofluorescence staining was conducted in at least two independent biological repeats.

**Colony formation and CCK8 assays**

Cells were seeded into 6-well plates (1.5×10^3^ cells/well) and left for 8-10 days until formation of visible colonies. Colonies were washed with PBS buffer, fixed with methanol for 30 min, and then stained with 0.1% crystal violet in 20% ethanol overnight. After staining, the plates were washed and air-dried. Eventually, the colony numbers were counted. Five independent experiments were performed for colony formation analysis.

For Cell Counting Kit-8 (CCK8) assay, cells (2.0×10^3^) were cultured in 96-well plates for 1 day, 2 days, 3 days and 4 days, respectively. Then, CCK8 solution (Beyotime, C0038) was added into each well in dark. Optical density (OD) values were determined by measuring the absorbance at 450 nm and 630 nm.

**Immunohistochemistry (IHC) staining**

The xenografted tumors were fixed in 4% formalin at room temperature for 48 h and embedded in paraffin, and the paraffin blocks were cut into 4-mm sections and deparaffinized. Routine IHC staining for Ki-67 (Cell Signaling Technology, #9449) was performed on the slides, then visualized by DAB Horseradish Peroxidase Color Development Kit (Beyotime, P0202). Apoptotic cells were identified by terminal deoxyribonucleotidyl transferase-mediated dUTP nick end labeling (TUNEL) staining according to the manufacturer's instructions (Beyotime, C1086).

**Reference**

1 Su Z, Sun Z, Wang Z, Wang S, Wang Y, Jin E *et al*. TIF1gamma inhibits lung adenocarcinoma EMT and metastasis by interacting with the TAF15/TBP complex. Cell Rep 2022; 41: 111513.
