# Supplementary material for: P4HA2 activates mTOR via hydroxylation and targeting P4HA2-mTOR inhibits lung adenocarcinoma cell growth
Source: Oncogene. 2024 Apr 23;43(24):1813–23. doi: 10.1038/s41388-024-03032-1 (PMC11164680; doi:10.1038/s41388-024-03032-1)
Supplement: Supplementary file 2 — Supplementary Figure Legends [file 41388_2024_3032_MOESM2_ESM.docx]

SUPPLEMENTARY FIGURE LEGENDS

Figure S1. Densitometric quantification of the immunoblot bands in Figures 1A, 2E, 2F, 3A-3D, 4D, and 6B. Immunoblotting experiments were performed in biological triplicates. Densitometric quantitation of various protein bands were from Figure 1A (A), Figure 2E (B), Figure 2F (C), Figure 3A (D), Figure 3B (E), Figure 3C (F), Figure 3D (G), Figure 4D (H), and Figure 6B (I). Densitometry values of phosphorylated proteins were normalized to those of total protein, mTOR hydroxylation levels were normalized to mTOR Co-IP products, and others were normalized to β-actin. The graphic data show the statistical results for densitometric quantitation of the immunoblot bands. Data are shown as means ± SD (n = 3). ns, not significant; **P*<0.05, ***P*<0.01, ****P*<0.001 by unpaired Student’s *t*-test.

Figure S2. Interaction between endogenous P4HA2 and mTOR in LUAD cells, and P4HA2 knockdown inhibits LUAD cell growth. (A) Co-IP was conducted in A549 cells. Endogenous P4HA2 or P4HB were immunoprecipitated with anti-mTOR antibody. Binding of P4HA2 or P4HB to mTOR was determined by immunoblot. (B) Analysis of mTOR mRNA expression in pan-cancer tissues and normal tissues using TCGA database. BRCA, Breast invasive carcinoma; CHOL, Cholangio carcinoma; ESCA, Esophageal carcinoma; HNSC, Head and neck squamous cell carcinoma; KIRC, Kidney renal clear cell carcinoma; KIRP, Kidney renal papillary cell carcinoma; LIHC, Liver hepatocellular carcinoma; LUAD, Lung Adenocarcinoma; THCA, Thyroid carcinoma; ns, not significant; **P* < 0.05, ***P* < 0.001 and ****P* < 0.001 by paired Student’s *t*-test. (C-F) Colony formation and CCK8 assays were performed in A549 and H1299 cells transfected with sh-P4HA2 or sh-NC. Data are shown as the mean ± SD (n = 5). ****P* < 0.001 by unpaired Student’s *t*-test.

Figure S3. EDHB inhibited mTOR protein expression and hydroxylation levels of total protein in A549 cells. A549 cells were treated with EDHB (200 μM) or DMSO (used as negative control). Whole cell lysates were immunoblotted with antibodies against pan-prolyl hydroxylation (Hy)-OH, mTOR, P4HA2 and β-actin as loading control. EDHB, ethyl-3,4-dihydroxybenzoate.

Figure S4. Hydroxylated P2341 electrostatically interacts with R2378 and thereby causing mTOR activation. (A) Comparison among the amino acid sequences within catalytic pockets of mTOR, SMG1 and CDK2. Positions of P2341/R2378, L2338/R2374 and P130/S188 were marked in the corresponding sequences as indicated. (B-E) Superimposition of mTOR (PDB:5H64) with SMG1 (PDB:6Z3R) and CDK2 (PDB:3QZW) showing potentially different contribution of P2341/R2378 to substrate recognition of mTOR when compared with those of L2338/R2374 and P130/S188 to substrate recognition of SMG1 and CDK2. (F) Serum-starved 293T cells co-transfected with Flag-mTOR^WT^ or Flag-mTOR^R2378A^ and HA-P4HA2 were stimulated with insulin (200 nM) for the indicated times. The WCL were immunoblotted with antibodies against Flag, HA, total or phosphorylated S6K and AKT.

Figure S5. mTOR^P2341A^ attenuates LUAD cell growth *in vivo*. (A) Tumor size analysis of the surgically excised tumors from individual BALB/c nude mice. A549 cells overexpressing mTOR^WT^, mTOR^P2341A^, and empty vector were subcutaneously implanted into BALB/c nude mice (5 mice per group). mTOR^WT^ mTOR wild-type; mTOR^P2341A^, mTOR mutant. (B and C) Changes in tumor volume (B) of the xenografts. Tumor size was measured using calipers every 3 days. Dot plot (C) showing changes in tumor volume after 15 days of treatment. Data are shown as the mean ± SD (n = 5); ****P* < 0.001 by unpaired Student’s *t*-test. (D and E) Immunohistochemistry staining of Ki67 (brown) and TUNEL (green) in subcutaneous tumors of BALB/c nude mice implanted with A549 cells overexpressing mTOR^WT^, mTOR^P2341A^, and empty vector. Cell proliferation (D) and apoptosis (E) were measured by Ki67 and TUNEL staining, respectively. Nuclei were counterstained with 4',6-diamidino-2-phenylindole (DAPI). Scale bar, 50 μm. Data are shown as the mean ± SD (n = 5); ****P* < 0.001 by unpaired Student’s *t*-test.

Figure S6. The contact of hydroxylated P2341 with prolines of mTOR substrates contributes to substrate recognition of mTOR. (A) Structure of a collagen triple-helix fragment composed of (Pro-Hyp-Gly)_n_ strands (PDB: 1CAG). (B-E) Superimposition of the substrate binding grooves of SMG1 (PDB: 6Z3R) and mTORC1 (PDB: 5H64) was visualized using the PyMol software, and 4E-BP1 substrate peptides containing P38 near T37, P47 near T46, P66 near S65 or P71 near T70 were overlaid into the catalytic pockets of mTORC1. This overlay yields the contact of mTOR P2341 with the prolines (P38, P47, P66, and P71) embodied in 4E-BP1 substrate peptides. (F and G) Superimposition of the substrate binding grooves of SMG1 (PDB: 6Z3R) and mTORC2 (PDB: 5ZCS) was visualized using the PyMol software, and PKC substrate peptides containing P635 near T631 or P639 near T638 were overlaid into the catalytic pockets of mTORC2. This overlay yields the contact of mTOR P2341 with the prolines (P635 and P639) embodied in PKC substrate peptide.

**Figure S7. Targeting P4HA2-mTOR inhibits LUAD cell growth.** (**A**) Schematic illustration of targeting P4HA2-mTOR through aspirin (potential P4HA2 inhibitor) and mTOR inhibitor AZD-8055. (**B** and **C**) A549 and H1299 cells were treated with DMSO, aspirin (1 mM), AZD-8055 (1 nM), and a combination of aspirin and AZD-8055 for 7 days and subjected to colony formation assays. Data are shown as the mean ± SD (n=5). ***P* < 0.01, ****P* < 0.001 by unpaired Student’s *t*-test. (**D**) Tumor size analysis of the surgically excised tumors from individual BALB/c nude mice. Mice bearing A549 xenograft tumors (4 mice per group) were randomized to four groups [vehicle, aspirin (100 mg/kg), AZD-8055 (10 mg/kg), and a combination of aspirin and AZD-8055]. (**E** and **F**) Tumor volume changes (E) of the xenografts following treatments with vehicle, aspirin, AZD-8055, and combined aspirin with AZD-8055. Tumor size was measured using calipers every 3 days. Dot plot (F) showing changes in tumor volume after 21 days of treatment. Data are shown as the mean ± SD (n = 4); ***P* < 0.01, ****P* < 0.001 by unpaired Student’s *t*-test.
