# Supplementary figures and images for: P4HA2 activates mTOR via hydroxylation and targeting P4HA2-mTOR inhibits lung adenocarcinoma cell growth
Source: Oncogene. 2024 Apr 23;43(24):1813–23. doi: 10.1038/s41388-024-03032-1 (PMC11164680; doi:10.1038/s41388-024-03032-1)

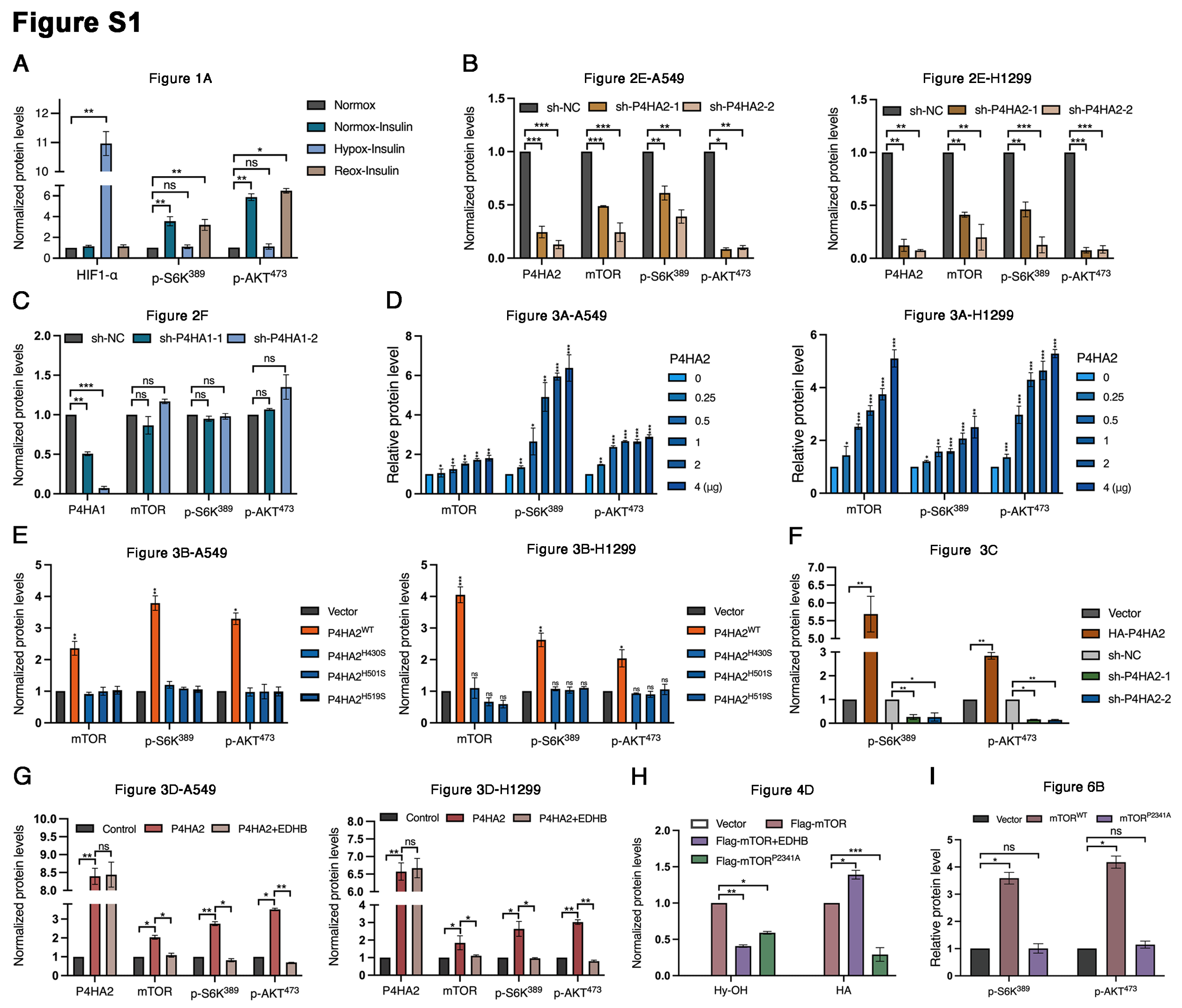

Supplement: Supplementary file 3 — Figure S1 [file 41388_2024_3032_MOESM3_ESM.tif]

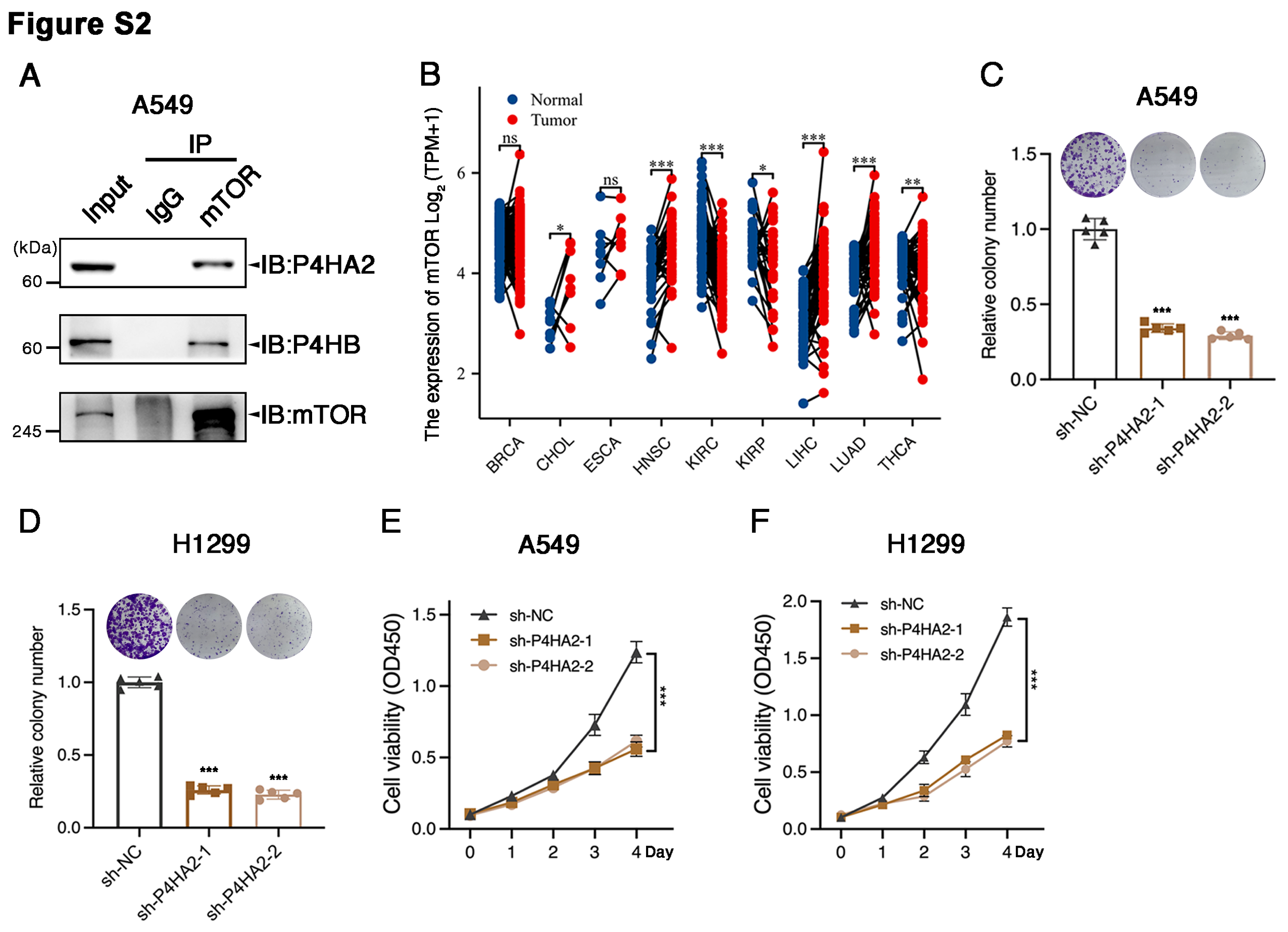

Supplement: Supplementary file 4 — Figure S2 [file 41388_2024_3032_MOESM4_ESM.tif]

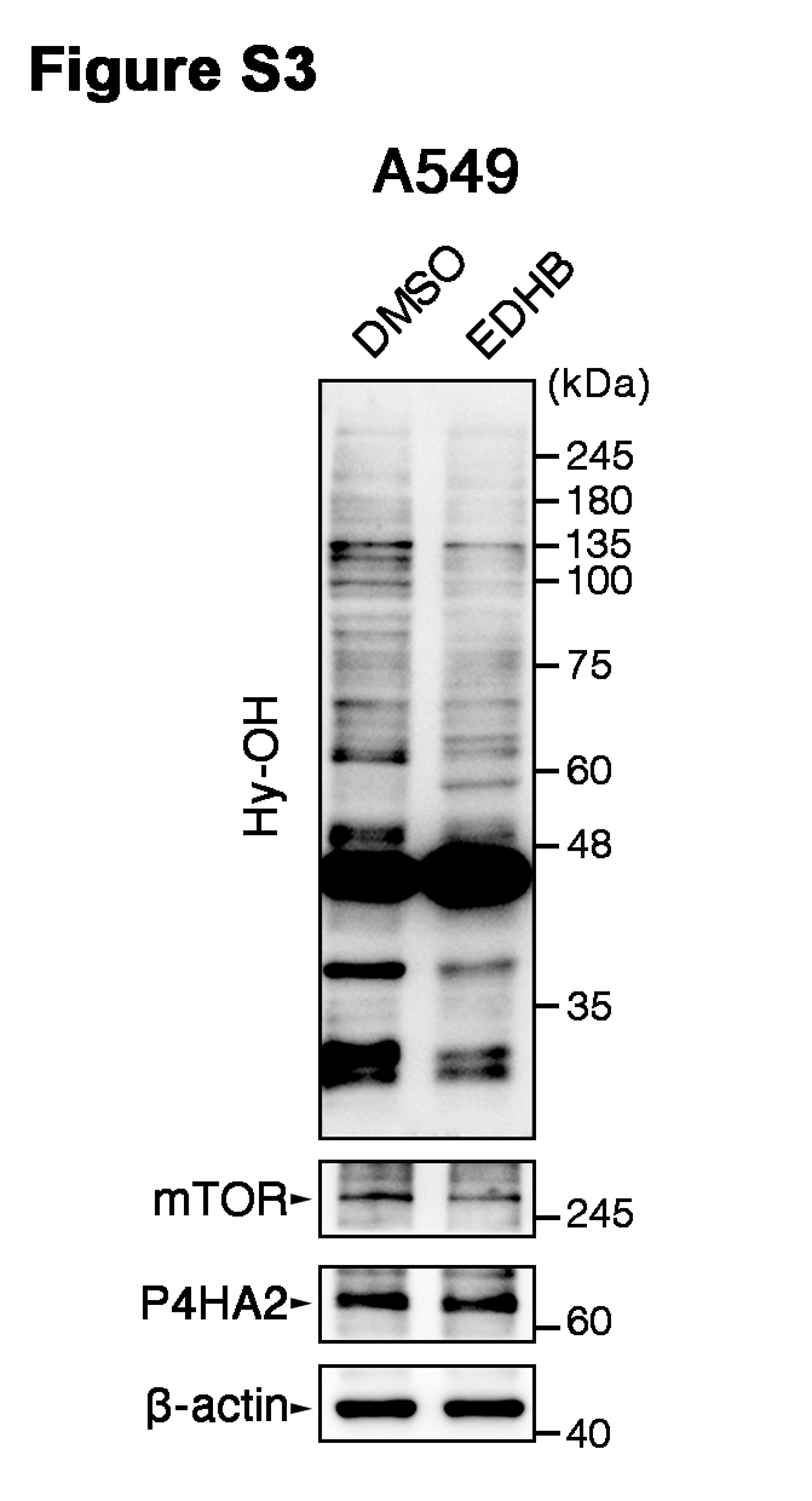

Supplement: Supplementary file 5 — Figure S3 [file 41388_2024_3032_MOESM5_ESM.tif]

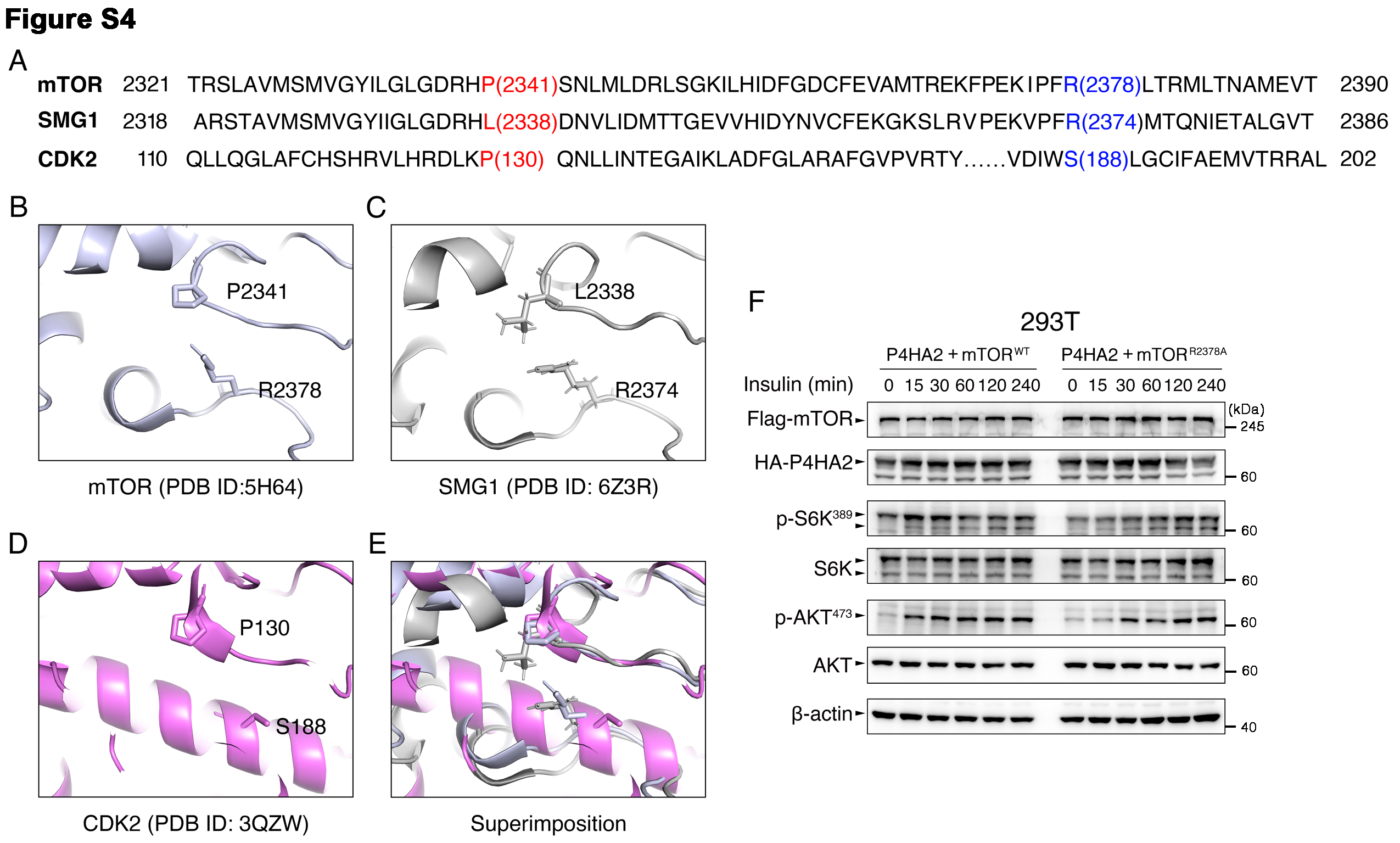

Supplement: Supplementary file 6 — Figure S4 [file 41388_2024_3032_MOESM6_ESM.tif]

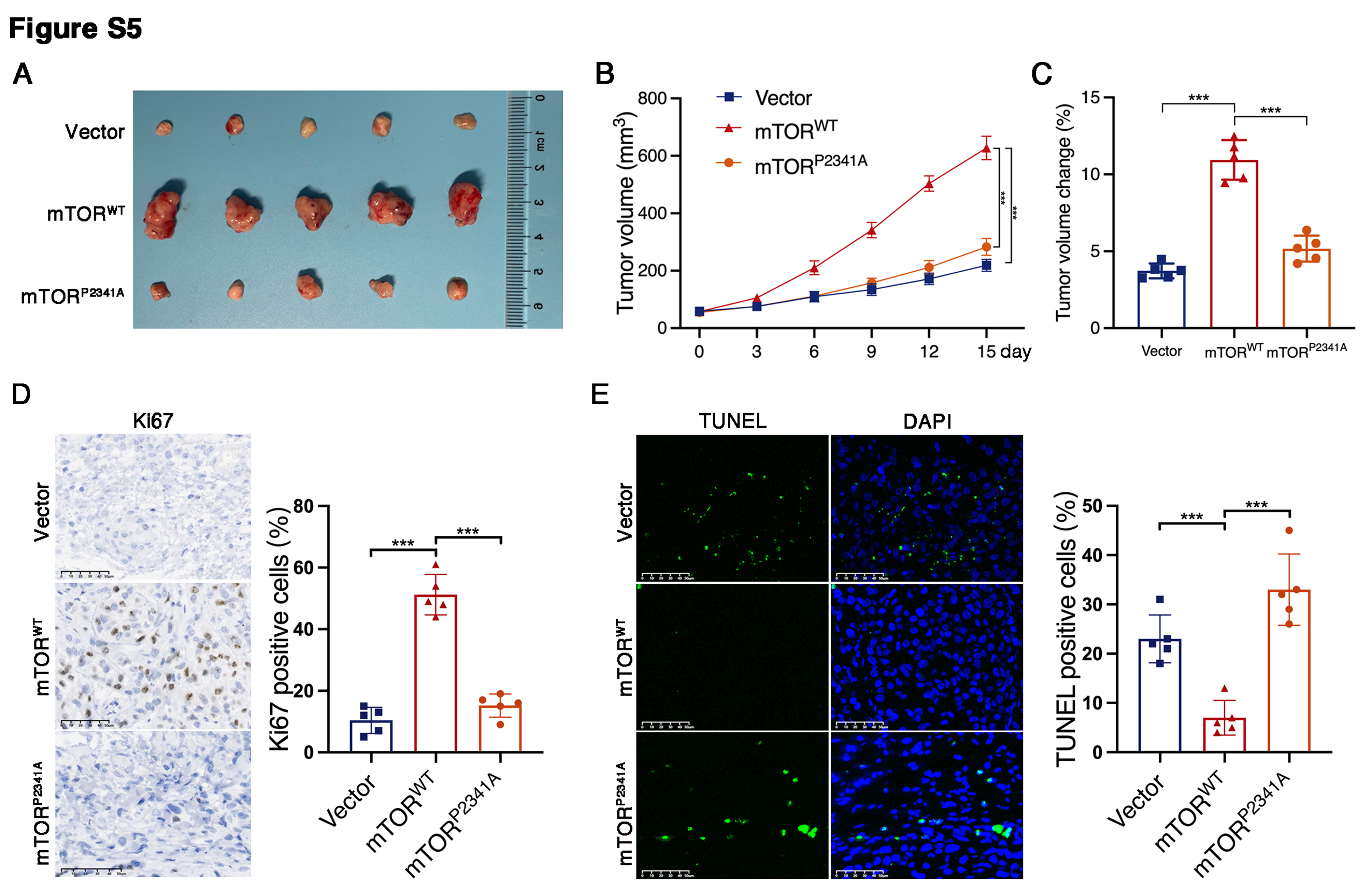

Supplement: Supplementary file 7 — Figure S5 [file 41388_2024_3032_MOESM7_ESM.tif]

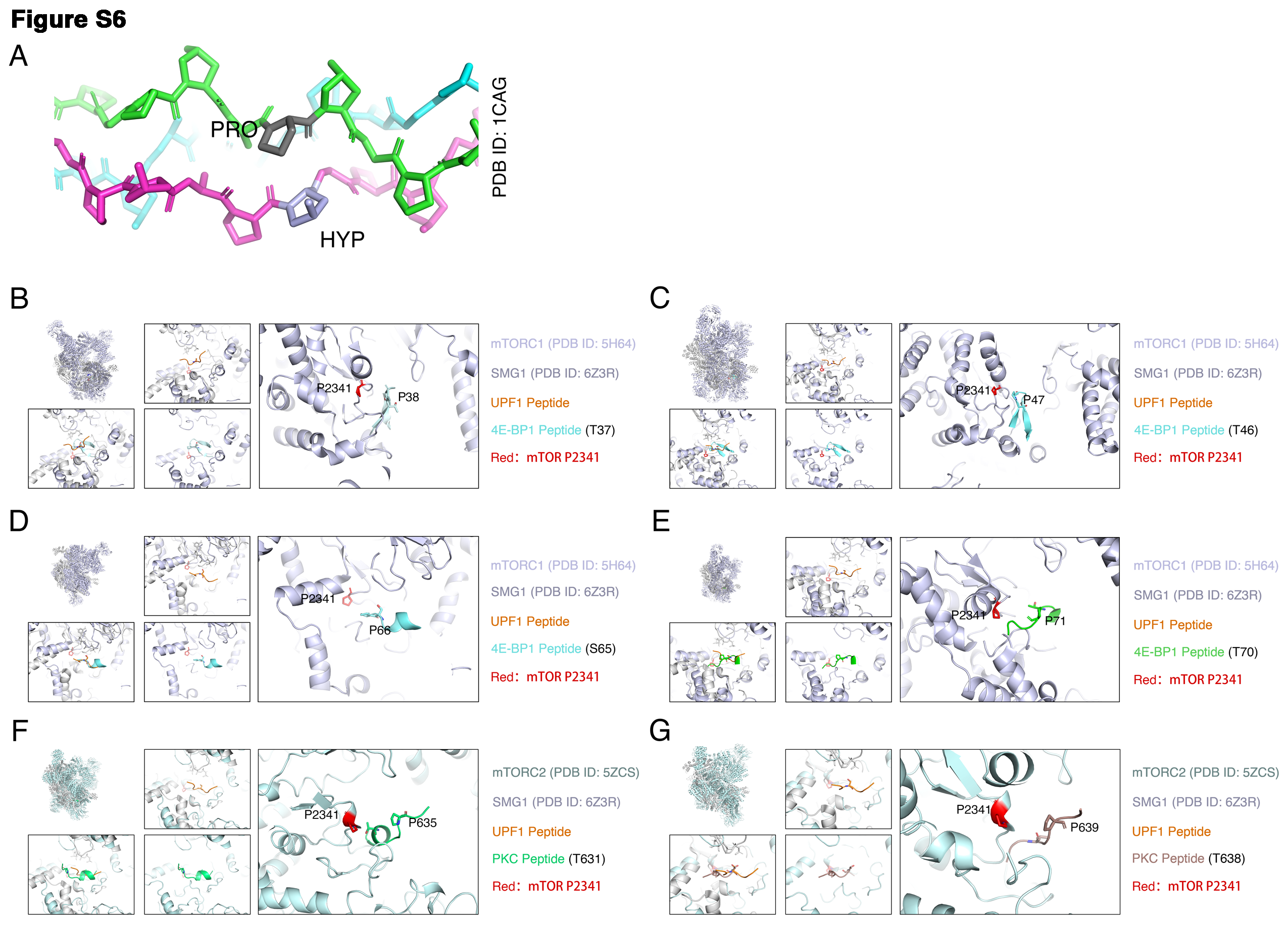

Supplement: Supplementary file 8 — Figure S6 [file 41388_2024_3032_MOESM8_ESM.tif]

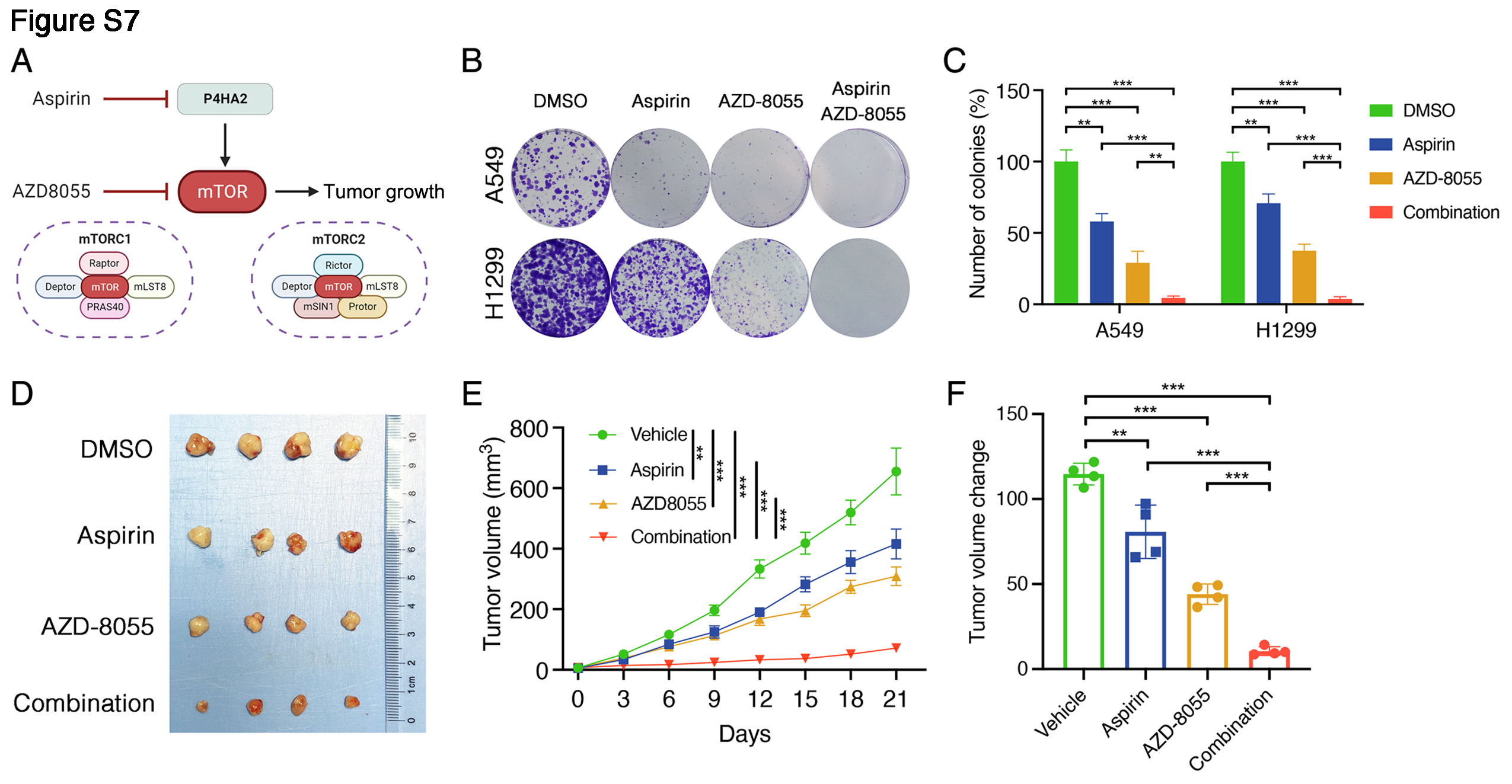

Supplement: Supplementary file 9 — Figure S7 [file 41388_2024_3032_MOESM9_ESM.tif]
